# Environmental benefits of switching from intravenous to oral administration of ciprofloxacin

**DOI:** 10.1093/jac/dkag163

**Published:** 2026-05-19

**Authors:** Nicolas De Jaegher, Julien De Greef, Caroline Briquet, Jean Cyr Yombi, Ysaline Toussaint, Patricia Luis, Hervé Jeanmart

**Affiliations:** Institute of Mechanics, Materials and Civil Engineering (iMMC), UCLouvain, Louvain-la-Neuve, Belgium; Departement of Internal Medicine and Infectious Diseases, Cliniques universitaires Saint-Luc, UCLouvain, Brussels, Belgium; Groupe de Gestion de l’Antibiothérapie, Cliniques universitaires Saint-Luc, UCLouvain, Brussels, Belgium; Louvain Centre for Toxicology and Applied Pharmacology, UCLouvain, Brussels, Belgium; Groupe de Gestion de l’Antibiothérapie, Cliniques universitaires Saint-Luc, UCLouvain, Brussels, Belgium; Department of Pharmacy, Cliniques universitaires Saint-Luc, UCLouvain, Brussels, Belgium; Departement of Internal Medicine and Infectious Diseases, Cliniques universitaires Saint-Luc, UCLouvain, Brussels, Belgium; Groupe de Gestion de l’Antibiothérapie, Cliniques universitaires Saint-Luc, UCLouvain, Brussels, Belgium; Institute of Mechanics, Materials and Civil Engineering (iMMC), UCLouvain, Louvain-la-Neuve, Belgium; Research and Innovation Centre for Process Engineering (ReCIPE), UCLouvain, Louvain-la-Neuve, Belgium; Institute of Mechanics, Materials and Civil Engineering (iMMC), UCLouvain, Louvain-la-Neuve, Belgium; Research and Innovation Centre for Process Engineering (ReCIPE), UCLouvain, Louvain-la-Neuve, Belgium; Institute of Mechanics, Materials and Civil Engineering (iMMC), UCLouvain, Louvain-la-Neuve, Belgium

Global warming represents a major threat to human health. The health care sector significantly contributes to this crisis, accounting for ∼4.4% of the global greenhouse gas emissions.^1^ Switching readily bioavailable drugs from intravenous (IV) to oral administration has been suggested as a way towards more sustainable practice, although supporting evidence remains scarce.^2^ Several antibiotics are suitable for IV to oral switch (IVOS) under appropriate clinical circumstances. IVOS has proven effective in multiple indications, including bacteraemia, osteoarticular infections and endocarditis, and is associated with improved safety, reduced costs and decreased nursing workload.^3,4^ We aimed to assess the environmental impact of IVOS by studying ciprofloxacin, an antibiotic with excellent bioavailability.

A life cycle assessment (LCA) of IV and oral ciprofloxacin administrations was performed following ISO 14040 and 14044 standards using the ecoinvent^®^ database v3.6 within the SimaPro^®^ software, quantifying their respective effects across 18 different categories, each focusing on a single environmental issue such as global warming, marine ecotoxicity or water consumption. The LCA adopted a cradle-to-grave approach encompassing: (i) active pharmaceutical ingredient (API) synthesis; (ii) production of the galenic formulation (i.e. the finished physical form of a drug produced by formulating one or more APIs with excipients); (iii) manufacturing of medical supplies and packaging; (iv) end-of-life (EoL) treatment for ciprofloxacin, medical supplies and packaging and (v) transport between life cycle stages. The scope of the LCA is detailed in Figure S1 (available as Supplementary data at *JAC* Online) and shipping distances are provided in Table S1. Packaging sterilization effects were not included in the analysis. In addition, catheter-related effects were also excluded, as a catheter is often inserted early in the clinical course and this analysis primarily aims at determining the impact of IVOS. The functional unit studied corresponds to one ciprofloxacin dose, i.e. 500 mg orally and 400 mg intravenously, including all related materials (Table S2 and Figure S2). Details on the data sources used in our LCA are provided in the Supplementary Methods.

Our primary analysis considered a standard clinical scenario where an IV line was maintained with 500 mL of normal saline daily for the purpose of IV antibiotic administration, i.e. the most effective situation for IVOS. Sensitivity analyses were performed for additional scenarios where: (i) the use of an IV line was shared with another drug administration; (ii) the maintenance of an IV line was requested independently of the antibiotics (in this scenario the impact of the IV line was not taken into account as IVOS would not lead to its removal) and (iii) variable maintenance fluid volumes. EoL treatment for medical supplies and packaging were assumed incinerated, and an additional scenario evaluated the impact of recycling on outcomes. Further details on methods and scenarios are provided as Supplementary Appendix.

IV administration demonstrated significantly higher environmental effects over oral administration across all 18 indicators, ranging from 20 times higher effects for marine eutrophication to 250 times higher effects for marine ecotoxicity (Tables S3–10, Figure S3). Regarding global warming, the carbon footprint of one oral dose was calculated at 12.6 gCO_2_ equivalent (gCO_2e_), whereas one IV dose reached almost 900 gCO_2e_ (Figure 1). In comparison, the ecoinvent^®^ database indicates that 1 km of travel by a medium-sized EURO5 diesel passenger car emits 321 gCO_2e_/km over its full life cycle.

**Figure 1.**
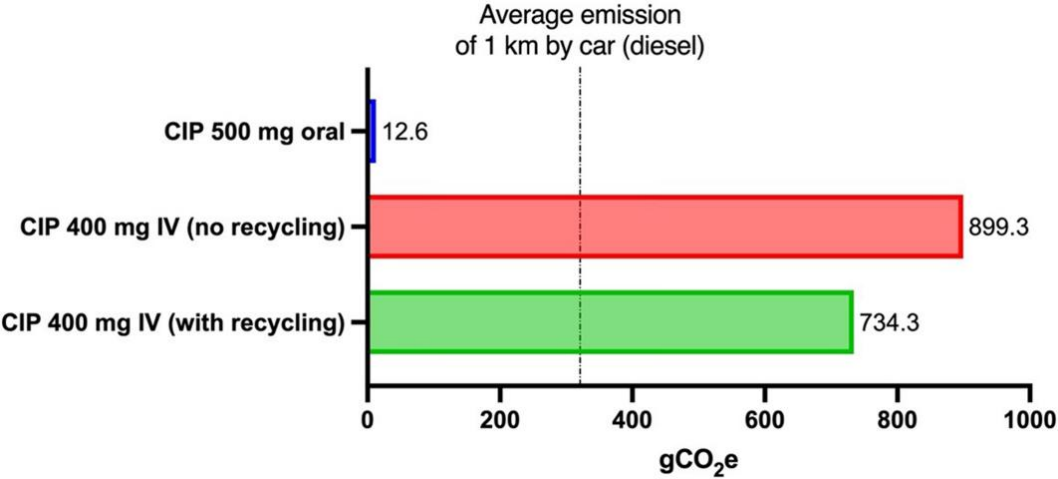
Greenhouse gas emissions of one dose of ciprofloxacin. IV emissions calculated under the primary clinical scenario where the IV line was only justified by antibiotic administration. Waste incineration was considered as EoL treatment for all materials, unless otherwise specified. The average life cycle greenhouse gas emissions of 1 km travelled by car are reported for comparison (321 gCO_2_e/km for a medium-sized diesel powered EURO5 transport passenger car; ecoinvent database). CIP, ciprofloxacin.

The production and EoL of medical supplies and related packaging accounted for most of the total impact across all factors. The IV solution packaging was found particularly significant, with its production and transport accounting for 216 gCO_2e_ in the primary IV scenario. By contrast, the contributions of API synthesis and galenic formulation were found negligible in the same scenario (respectively, 4.4 gCO_2e_ and 0.8 gCO_2e_). Further analysis of additional scenarios revealed only modest reductions in environmental effects from waste recycling: for example, an 18.3% decrease in CO_2e_ emissions related to IV administration. The results also highlighted that reducing maintenance fluid volumes, when clinically feasible, could partially offset the environmental footprint of IV administration, as illustrated by a 26.0% reduction in CO_2e_ emissions for 250 mL compared with 1000 mL (Supplementary Results).

Our results offer the first rigorous demonstration of the significant environmental benefits of IVOS for antibiotics, focusing on ciprofloxacin. Clindamycin, metronidazole, tetracyclines, co-trimoxazole, azoles, linezolid as well as other fluoroquinolones are also well-known good candidates for IVOS.^3^ Although our results cannot be directly translated to these agents, the magnitude of the impact difference, the limited API synthesis contribution, as well as comparable findings previously reported for acetaminophen and ketoprophen IVOS, support broader relevance.^5,6^ A limitation of this work is the exclusion of packaging sterilization effects, although existing data suggest these are minor and higher for IV compared with oral formulation.^6^ Overall, our findings introduce and quantify the substantial environmental co-benefits of antibiotics IVOS, complementing existing evidence that supports its use in appropriate clinical circumstances, balancing patient safety, comfort and cost-effectiveness.

## Supplementary Material

dkag163_Supplementary_Data

## Supplementary data

Figures S1 –S3 and Tables S1–S10 are available as Supplementary data at *JAC* Online.
