# Supplementary material for: Environmental benefits of switching from intravenous to oral administration of ciprofloxacin
Source: J Antimicrob Chemother. 2026 May 19;81(6):dkag163. doi: 10.1093/jac/dkag163 (PMC13184615; doi:10.1093/jac/dkag163)
Supplement: dkag163_Supplementary_Data [file dkag163_supplementary_data.docx]

# Environmental Benefits of Switching from Intravenous to Oral Administration of Ciprofloxacin - Supplementary material

### Supplementary Methods

Generalities

The life cycle analysis (LCA) was performed following ISO 14040 and 14044 standards using ecoinvent® database v3.6 within the SimaPro® software environment and applies the ReCiPe 2016 Midpoint (H) method. H stands for hierarchist perspective, which reflects a scientific consensus and a balanced, medium-term view of environmental impacts. Midpoint stands for impact categories measured at an intermediate stage in the environmental cause-effect chain. This work covers 18 different impact categories: global warming (g CO₂ eq); stratospheric ozone depletion (g CFC11 eq); ionizing radiation (Bq Co-60 eq); ozone formation, human health (g NOₓ eq); fine particulate matter formation (g PM2.5 eq); ozone formation, terrestrial ecosystems (g NOₓ eq); terrestrial acidification (g SO₂ eq); freshwater eutrophication (g P eq); marine eutrophication (g N eq); terrestrial ecotoxicity (g 1,4-DCB); freshwater ecotoxicity (g 1,4-DCB); marine ecotoxicity (g 1,4-DCB); human carcinogenic toxicity (g 1,4-DCB); human non-carcinogenic toxicity (g 1,4-DCB); land use (m²a crop eq); mineral resource scarcity (g Cu eq); fossil resource scarcity (g oil eq); water consumption (L).

The following data sources were used in the LCA: (i) for API production: results from Yang et al. (2020), from where energy, chemical products, water consumption, gas liberated, waste generated and waste water were integrated in the simulation^1^; (ii) for galenic formulations: pharmaceutical notices, industry data, and literature^2, 3^; (iii) for packaging and supplies compositions: medical supply notices or FTIR analysis when information was not available, with components being disassembled and weighed in the laboratory; (iv) Usetox was applied to characterize ciprofloxacin’s freshwater ecotoxicity after the patient excretes the antibiotic into the wastewater.^4, 5^

All impacts were calculated for an antibiotic administered at Cliniques universitaires Saint-Luc, Brussels, Belgium. In our simulation, it was chosen to use the following modes of transport between the last factories from each material and the Cliniques universitaires Saint-Luc: "Transport, freight, lorry 16-32 metric ton, euro6 RER| market for transport, freight, lorry 16-32 metric ton, EURO6 | APOS, S". Table S1 provides the distances between those different stages of the life cycle. Transport was also included between API production and the factory that produces the galenic formulation (East Asia to East Europe).

Figure 1 was created with Prism 10 (v10.6) for MacOS. Figure S2 was created with Microsoft Excel (v16.78.3).

Functional unit

The functional unit in this analysis is defined as one standard dose of ciprofloxacin, i.e., 500 mg for oral administration and 400 mg for intravenous (IV) aqueous solution administration. The details of all materials included in this LCA are provided in Table S2.

The environmental impact of the medical supplies for IV administration was assessed according to their functional lifetime per administration, excluding the catheter, as a catheter is often inserted early in the clinical course and this analysis primarily aims at determining the impact of IVOS. As an IV administration requires equipments that are not changed with each administration, Table S2 shows the fraction of each compound considered in our analysis for one functional unit, according to local infection control guidelines. The lifetime in number of doses by medical supply corresponds to the amount of time the medical supply is used before being changed. For example, as the maintenance infusion bag is replaced once per day, its lifetime is 2 doses of ciprofloxacin.

For oral administration, the outer packaging (Calypso OS, Synteco) was considered per dose in addition to the tablet in its primary blister packaging.

LCA boundaries and limits

The LCA adopted a cradle-to-grave approach (Figure S1), encompassing: (i) active pharmaceutical ingredients (API) synthesis; (ii) galenic formulation production; (iii) manufacturing of medical supplies, and packaging for these items and the drug; (iv) end-of-life (EoL) treatment for ciprofloxacin, medical supplies, and packaging; (v) transport between life cycle stages.

The impact of API and galenic formulation sterilization were included in the analysis, based on published data^1, 2^. On the contrary, sterilization of packaging and line-related supplies was excluded from the analysis as detailed information about used sterilization techniques and procedures were not available. Of note, the estimated impact for greenhouse gases (and possibly other impacts) is expected to be limited based on the results from a LCA for paracetamol administration in which sterilization was included and was found to be by far the least impactful element of the carbon footprint.^6^

Impacts associated with catheter and its placement were excluded from the analysis, as a catheter is often inserted early in the clinical course and this analysis primarily aims at determining the impact of IV to oral shift.

**Supplementary Table 1. Detail of materials included in the analysis for IV ciprofloxacin administration.**

| **Medical Supplies and Medications** | **Name of Medical Supply** | **Brand** | **Shipping distance from last factory (km)** |
| --- | --- | --- | --- |
| CIP in injectable form | Ciprofloxacin Fresenius KabiPac^®^ 400 mg/200 mL | Fresenius Kabi | 1300 |
| Maintenance infusion bag | Viaflo^®^ NaCl 0.9% 500 mL | Baxter | 80 |
| IV infusion set | Intrafix^®^ Safeset | B.Braun | 450 |
| Flow rate controller | Dosicair^®^ DF050 | Cair LGL | 450 |
| Manifold (3 port) | Manifold RP3000M | Cair LGL | 450 |
| Extension line | Extension line M/F 85 cm | Sasan | 3100 |
| Disinfectant | Chlorhexidini Gluconas 2% - Ethylalcohol 70% (250 mL) | Magis Pharma | 50 |
| Sterile compress | Sterilux^®^ ES | Hartmann | 600 |
| CIP as tablet | Ciprofloxacin Sandoz 500 mg | Sandoz | 3200 |
| Pouch secondary packaging^1^ | Calypso OS | Synteco Robotics & automation | 0 |

Shipping distances were calculated based on the location of Cliniques universitaires Saint-Luc (Woluwé-Saint-Lambert, Belgium) and the producing factory, as referenced on the product’s notice or the producer’s website.

^1^Cliniques universitaires Saint-Luc uses an automated system to dispense medications in unit doses. Each Ciproxine tablet, kept in its original primary packaging, is overwrapped in a Calypso OS pouch compatible with the Pegasus dispensing machine (Synteco). This paper-plastic packaging includes a QR code (batch number, expiration date, etc.) ensuring full traceability up to patient administration.

**Supplementary Table 2. Description of medical supplies per functional unit for oral and IV ciprofloxacin.**

| **Medical supply** | **Description of local recommendations and/or use** | **Recommended quantity per day** | **Quantity per functional unit** |
| --- | --- | --- | --- |
| ***Oral administration*** | | | |
| Ciprofloxacin tablet 500 mg | Standard oral dosage  500mg 2x/d | 2 | 1 |
| Pouch packaging (Calypso OS) | Specific hospital packaging (for automated delivery by Pegasus dispensing machine and traceability up to patient administration) | 2 | 1 |
| ***IV administration*** | | | |
| Ciprofloxacin 400 mg (KabiPac^®^) | Standard IV dosage  400mg 2x/d | 2 | 1 |
| IV infusion set (connected to antibiotic) | Local hospital hygiene recommendations: 1/24h | 1 | 0.5 |
| Maintenance infusion bag | Standard maintenance infusion  500 ml normal saline /day | 1 | 0.5 |
| IV infusion set (connected to maintenance infusion bag) | Local hospital hygiene recommendations: 1/96h | 0.25 | 0.125 |
| Flow rate controller | Local hospital hygiene recommendations: 1/96h | 0.25 | 0.125 |
| Manifold  (3 port) | Local hospital hygiene recommendations: 1/96h | 0.25 | 0.125 |
| Extension line | Local hospital hygiene recommendations: 1/96h | 0.25 | 0.125 |
| Disinfectant  (10 mL) | Estimation of 10 mL used per patient per day related to IV administrations | 1 | 0.5 |
| Sterile compresses | Estimation of 1 pack of 5 compresses per patient per day related to IV administrations | 1 | 0.5 |

**Supplementary Figure 1. Description of the scope of the LCA.**

**
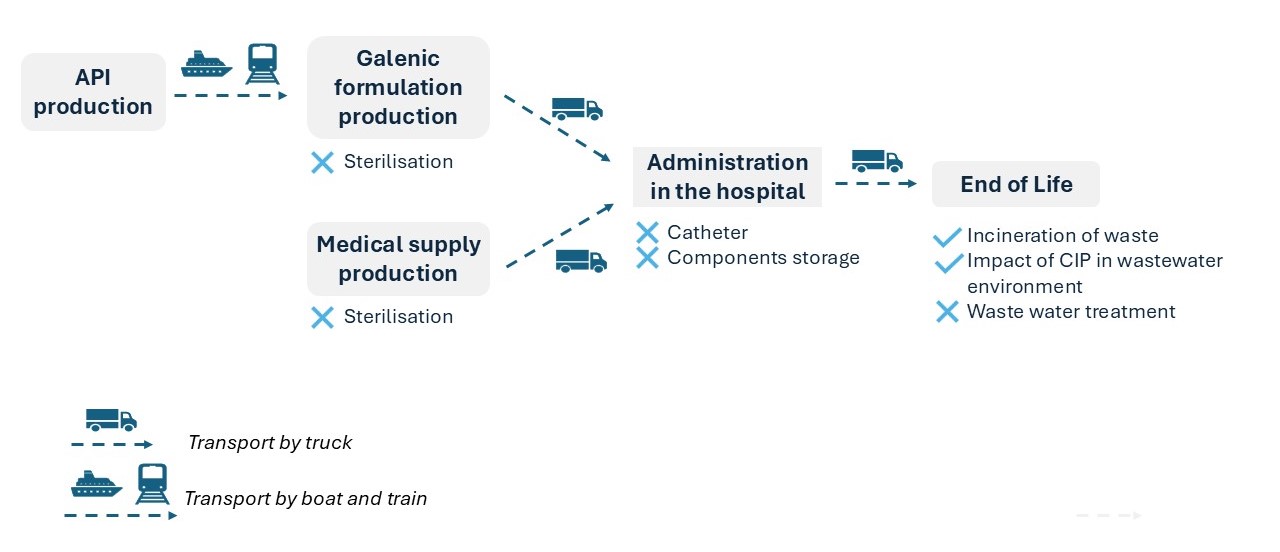
**

**Supplementary Figure 2.** Details of materials included in the analysis for the main scenario of IV (1 to 8) and oral (9) ciprofloxacin administration. The figure constituents are illustrative of materials included in the analysis but do not correspond to one functional unit (see Table S2 for quantities defining one functional unit).

| 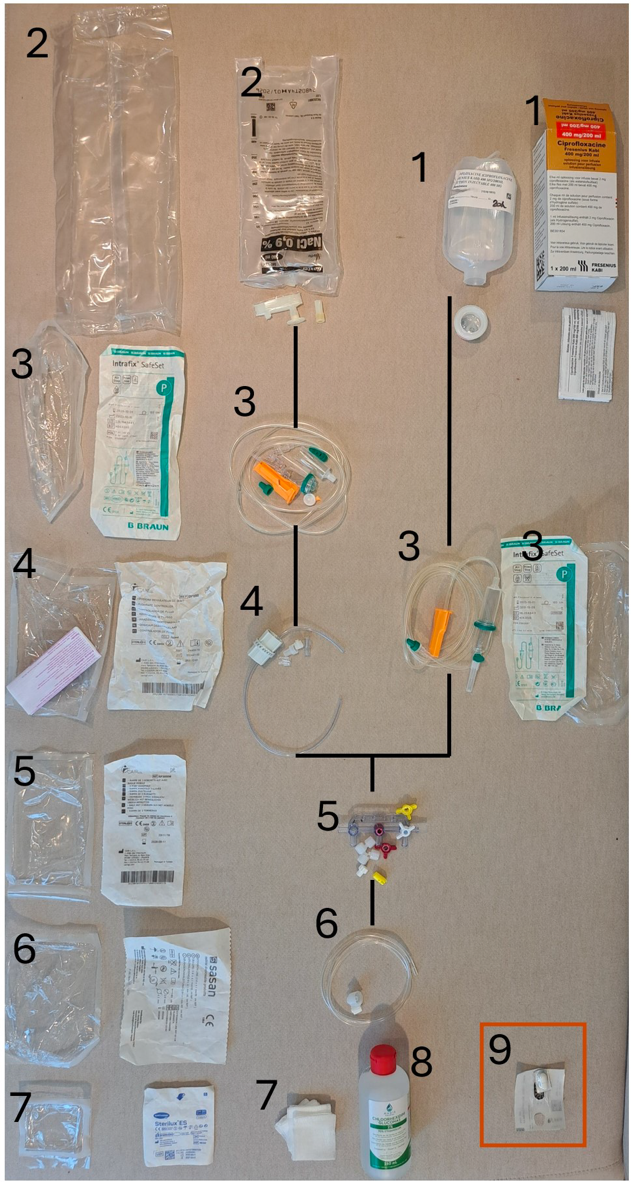 | 1. 400 mg of ciprofloxacin in injectable form and its primary packaging (left), secondary packaging and notice (right).  2. Maintenance infusion bag of 500 mL (main scenario): secondary packaging (left), physiological liquid and  primary packaging (right).  3. IV infusion set: primary packaging (left and right), IV infusion set (middle).  4. Flow rate controller: primary packaging and notice (left), flow rate controller  (right).  5. Manifold (3-port): primary packaging (left), manifold (3-port) (right).  6. Extension line: primary packaging (left), extension line (right).  7. Sterile compress: primary packaging (left), sterile compress (right).  8. Disinfectant and primary packaging.  9: Reference flow of the oral ciprofloxacin. In the orange rectangle: a tablet, a blister, and a pouch. |
| --- | --- |

### Supplementary Results

**Supplementary Table 3. Detailed environmental impacts across 18 different categories for one dose of ciprofloxacin administered IV (400 mg) - main scenario.** Impacts are subcategorized into their four components: related to the API, the galenic formulation, the medical supply and packaging necessary for administration and the waste treatment. Abbreviations: API: Active Pharmaceutical Ingredient.

| **Impact category** | **Unit** | **Total** | **API** | **Galenic** | **Medical supply** | **Waste treatment** |
| --- | --- | --- | --- | --- | --- | --- |
| Global warming | kg CO2 eq | 8,99E-01 | 4,37E-03 | 8,05E-04 | 4,71E-01 | 4,23E-01 |
| Stratospheric ozone depletion | kg CFC11 eq | 1,09E-06 | 1,63E-09 | 5,91E-10 | 7,83E-07 | 3,07E-07 |
| Ionizing radiation | kBq Co-60 eq | 1,96E-01 | 1,75E-04 | 7,13E-05 | 1,95E-01 | 5,50E-04 |
| Ozone formation, Human health | kg NOx eq | 1,26E-03 | 7,85E-06 | 6,05E-06 | 9,90E-04 | 2,51E-04 |
| Fine particulate matter formation | kg PM2.5 eq | 6,54E-04 | 5,25E-06 | 2,99E-06 | 5,94E-04 | 5,13E-05 |
| Ozone formation, Terrestrial ecosystems | kg NOx eq | 1,31E-03 | 8,22E-06 | 6,11E-06 | 1,05E-03 | 2,55E-04 |
| Terrestrial acidification | kg SO2 eq | 1,54E-03 | 1,16E-05 | 7,95E-06 | 1,39E-03 | 1,33E-04 |
| Freshwater eutrophication | kg P eq | 3,31E-04 | 5,75E-06 | 4,04E-07 | 1,93E-04 | 1,31E-04 |
| Marine eutrophication | kg N eq | 1,06E-04 | 3,72E-06 | 2,72E-08 | 9,56E-05 | 6,66E-06 |
| Terrestrial ecotoxicity | kg 1,4-DCB | 2,68E+00 | 1,21E-02 | 7,57E-03 | 2,47E+00 | 1,90E-01 |
| Freshwater ecotoxicity | kg 1,4-DCB | 1,60E-01 | 1,44E-04 | 7,99E-05 | 2,35E-02 | 1,36E-01 |
| Marine ecotoxicity | kg 1,4-DCB | 2,10E-01 | 1,90E-04 | 1,05E-04 | 3,22E-02 | 1,78E-01 |
| Human carcinogenic toxicity | kg 1,4-DCB | 4,20E-02 | 3,09E-04 | 9,30E-05 | 2,42E-02 | 1,75E-02 |
| Human non-carcinogenic toxicity | kg 1,4-DCB | 3,20E+00 | 3,14E-03 | 1,46E-03 | 7,48E-01 | 2,45E+00 |
| Land use | m2a crop eq | 6,38E-02 | 6,96E-05 | 2,96E-05 | 6,32E-02 | 4,77E-04 |
| Mineral resource scarcity | kg Cu eq | 2,26E-03 | 9,22E-06 | 6,79E-06 | 2,10E-03 | 1,43E-04 |
| Fossil resource scarcity | kg oil eq | 1,84E-01 | 1,45E-03 | 2,17E-04 | 1,76E-01 | 6,56E-03 |
| Water consumption | m3 | 2,08E-02 | 6,92E-05 | 1,49E-04 | 1,99E-02 | 6,83E-04 |

**Supplementary Table 4. Detailed environmental impacts across 18 different categories for one dose of ciprofloxacin administered IV (400 mg) - alternative scenario: the use of an IV line shared with another drug administration.** Impacts are subcategorized into their four components: related to the API, the galenic formulation, the medical supply and packaging necessary for administration and the waste treatment. Abbreviations: API: Active Pharmaceutical Ingredient.

| **Impact category** | **Unit** | **Total** | **API** | **Galenic** | **Medical supply** | **Waste treatment** |
| --- | --- | --- | --- | --- | --- | --- |
| Global warming | kg CO2 eq | 7,14E-01 | 4,37E-03 | 8,05E-04 | 3,86E-01 | 3,23E-01 |
| Stratospheric ozone depletion | kg CFC11 eq | 7,14E-07 | 1,63E-09 | 5,91E-10 | 4,89E-07 | 2,23E-07 |
| Ionizing radiation | kBq Co-60 eq | 1,68E-01 | 1,75E-04 | 7,13E-05 | 1,68E-01 | 4,12E-04 |
| Ozone formation, Human health | kg NOx eq | 1,02E-03 | 7,85E-06 | 6,05E-06 | 8,05E-04 | 1,99E-04 |
| Fine particulate matter formation | kg PM2.5 eq | 5,37E-04 | 5,25E-06 | 2,99E-06 | 4,88E-04 | 4,07E-05 |
| Ozone formation, Terrestrial ecosystems | kg NOx eq | 1,07E-03 | 8,22E-06 | 6,11E-06 | 8,50E-04 | 2,02E-04 |
| Terrestrial acidification | kg SO2 eq | 1,24E-03 | 1,16E-05 | 7,95E-06 | 1,11E-03 | 1,05E-04 |
| Freshwater eutrophication | kg P eq | 2,64E-04 | 5,75E-06 | 4,04E-07 | 1,59E-04 | 9,87E-05 |
| Marine eutrophication | kg N eq | 6,48E-05 | 3,72E-06 | 2,72E-08 | 5,62E-05 | 4,84E-06 |
| Terrestrial ecotoxicity | kg 1,4-DCB | 2,38E+00 | 1,21E-02 | 7,57E-03 | 2,22E+00 | 1,41E-01 |
| Freshwater ecotoxicity | kg 1,4-DCB | 1,19E-01 | 1,44E-04 | 7,99E-05 | 1,98E-02 | 9,87E-02 |
| Marine ecotoxicity | kg 1,4-DCB | 1,57E-01 | 1,90E-04 | 1,05E-04 | 2,74E-02 | 1,29E-01 |
| Human carcinogenic toxicity | kg 1,4-DCB | 3,36E-02 | 3,09E-04 | 9,30E-05 | 2,05E-02 | 1,27E-02 |
| Human non-carcinogenic toxicity | kg 1,4-DCB | 2,43E+00 | 3,14E-03 | 1,46E-03 | 6,38E-01 | 1,79E+00 |
| Land use | m2a crop eq | 5,26E-02 | 6,96E-05 | 2,96E-05 | 5,21E-02 | 3,79E-04 |
| Mineral resource scarcity | kg Cu eq | 1,92E-03 | 9,22E-06 | 6,79E-06 | 1,79E-03 | 1,06E-04 |
| Fossil resource scarcity | kg oil eq | 1,52E-01 | 1,45E-03 | 2,17E-04 | 1,45E-01 | 5,39E-03 |
| Water consumption | m3 | 1,51E-02 | 6,92E-05 | 1,49E-04 | 1,44E-02 | 5,02E-04 |

**Supplementary Table 5. Detailed environmental impacts across 18 different categories for one dose of ciprofloxacin administered IV (400 mg) - alternative scenario: the use of a maintenance fluid of 250 mL.** Impacts are subcategorized into their four components: related to the API, the galenic formulation, the medical supply and packaging necessary for administration and the waste treatment. Abbreviations: API: Active Pharmaceutical Ingredient.

| **Impact category** | **Unit** | **Total** | **API** | **Galenic** | **Medical supply** | **Waste treatment** |
| --- | --- | --- | --- | --- | --- | --- |
| Global warming | kg CO2 eq | 8,02E-01 | 4,37E-03 | 8,05E-04 | 4,50E-01 | 3,47E-01 |
| Stratospheric ozone depletion | kg CFC11 eq | 8,97E-07 | 1,63E-09 | 5,91E-10 | 6,58E-07 | 2,37E-07 |
| Ionizing radiation | kBq Co-60 eq | 1,95E-01 | 1,75E-04 | 7,13E-05 | 1,95E-01 | 4,40E-04 |
| Ozone formation, Human health | kg NOx eq | 1,17E-03 | 7,85E-06 | 6,05E-06 | 9,46E-04 | 2,08E-04 |
| Fine particulate matter formation | kg PM2.5 eq | 6,24E-04 | 5,25E-06 | 2,99E-06 | 5,73E-04 | 4,25E-05 |
| Ozone formation, Terrestrial ecosystems | kg NOx eq | 1,23E-03 | 8,22E-06 | 6,11E-06 | 9,99E-04 | 2,12E-04 |
| Terrestrial acidification | kg SO2 eq | 1,46E-03 | 1,16E-05 | 7,95E-06 | 1,33E-03 | 1,09E-04 |
| Freshwater eutrophication | kg P eq | 2,99E-04 | 5,75E-06 | 4,04E-07 | 1,88E-04 | 1,04E-04 |
| Marine eutrophication | kg N eq | 1,04E-04 | 3,72E-06 | 2,72E-08 | 9,48E-05 | 5,18E-06 |
| Terrestrial ecotoxicity | kg 1,4-DCB | 2,59E+00 | 1,21E-02 | 7,57E-03 | 2,42E+00 | 1,45E-01 |
| Freshwater ecotoxicity | kg 1,4-DCB | 1,24E-01 | 1,44E-04 | 7,99E-05 | 2,32E-02 | 1,01E-01 |
| Marine ecotoxicity | kg 1,4-DCB | 1,64E-01 | 1,90E-04 | 1,05E-04 | 3,19E-02 | 1,32E-01 |
| Human carcinogenic toxicity | kg 1,4-DCB | 3,71E-02 | 3,09E-04 | 9,30E-05 | 2,36E-02 | 1,31E-02 |
| Human non-carcinogenic toxicity | kg 1,4-DCB | 2,58E+00 | 3,14E-03 | 1,46E-03 | 7,41E-01 | 1,83E+00 |
| Land use | m2a crop eq | 6,35E-02 | 6,96E-05 | 2,96E-05 | 6,30E-02 | 3,91E-04 |
| Mineral resource scarcity | kg Cu eq | 2,21E-03 | 9,22E-06 | 6,79E-06 | 2,08E-03 | 1,10E-04 |
| Fossil resource scarcity | kg oil eq | 1,75E-01 | 1,45E-03 | 2,17E-04 | 1,68E-01 | 5,54E-03 |
| Water consumption | m3 | 2,04E-02 | 6,92E-05 | 1,49E-04 | 1,97E-02 | 5,25E-04 |

**Supplementary Table 6. Detailed environmental impacts across 18 different categories for one dose of ciprofloxacin administered IV (400 mg) - alternative scenario: the use of a maintenance fluid of 1000 mL.** Impacts are subcategorized into their four components: related to the API, the galenic formulation, the medical supply and packaging necessary for administration and the waste treatment. Abbreviations: API: Active Pharmaceutical Ingredient.

| **Impact category** | **Unit** | **Total** | **API** | **Galenic** | **Medical supply** | **Waste treatment** |
| --- | --- | --- | --- | --- | --- | --- |
| Global warming | kg CO2 eq | 1,08E+00 | 4,37E-03 | 8,05E-04 | 5,13E-01 | 5,66E-01 |
| Stratospheric ozone depletion | kg CFC11 eq | 1,34E-06 | 1,63E-09 | 5,91E-10 | 8,80E-07 | 4,53E-07 |
| Ionizing radiation | kBq Co-60 eq | 2,49E-01 | 1,75E-04 | 7,13E-05 | 2,48E-01 | 7,72E-04 |
| Ozone formation, Human health | kg NOx eq | 1,45E-03 | 7,85E-06 | 6,05E-06 | 1,10E-03 | 3,40E-04 |
| Fine particulate matter formation | kg PM2.5 eq | 7,36E-04 | 5,25E-06 | 2,99E-06 | 6,58E-04 | 6,94E-05 |
| Ozone formation, Terrestrial ecosystems | kg NOx eq | 1,52E-03 | 8,22E-06 | 6,11E-06 | 1,16E-03 | 3,44E-04 |
| Terrestrial acidification | kg SO2 eq | 1,74E-03 | 1,16E-05 | 7,95E-06 | 1,53E-03 | 1,82E-04 |
| Freshwater eutrophication | kg P eq | 4,08E-04 | 5,75E-06 | 4,04E-07 | 2,17E-04 | 1,85E-04 |
| Marine eutrophication | kg N eq | 1,14E-04 | 3,72E-06 | 2,72E-08 | 1,01E-04 | 9,73E-06 |
| Terrestrial ecotoxicity | kg 1,4-DCB | 3,11E+00 | 1,21E-02 | 7,57E-03 | 2,81E+00 | 2,83E-01 |
| Freshwater ecotoxicity | kg 1,4-DCB | 2,36E-01 | 1,44E-04 | 7,99E-05 | 2,81E-02 | 2,08E-01 |
| Marine ecotoxicity | kg 1,4-DCB | 3,10E-01 | 1,90E-04 | 1,05E-04 | 3,87E-02 | 2,71E-01 |
| Human carcinogenic toxicity | kg 1,4-DCB | 5,44E-02 | 3,09E-04 | 9,30E-05 | 2,77E-02 | 2,63E-02 |
| Human non-carcinogenic toxicity | kg 1,4-DCB | 4,62E+00 | 3,14E-03 | 1,46E-03 | 9,12E-01 | 3,71E+00 |
| Land use | m2a crop eq | 7,40E-02 | 6,96E-05 | 2,96E-05 | 7,32E-02 | 6,53E-04 |
| Mineral resource scarcity | kg Cu eq | 2,81E-03 | 9,22E-06 | 6,79E-06 | 2,58E-03 | 2,12E-04 |
| Fossil resource scarcity | kg oil eq | 2,00E-01 | 1,45E-03 | 2,17E-04 | 1,90E-01 | 8,67E-03 |
| Water consumption | m3 | 2,42E-02 | 6,92E-05 | 1,49E-04 | 2,29E-02 | 1,01E-03 |

**Supplementary Table 7. Detailed environmental impacts across 18 different categories for one dose of ciprofloxacin administered IV (400 mg) - alternative scenario: impact associated exclusively with the 400 mg IV ciprofloxacin.** This scenario represents the case when the maintenance of an IV line is requested in case of IVOS: the impact of the IV line was not taken into account as IVOS would not lead to its removal. Impacts are subcategorized into their four components: related to the API, the galenic formulation, the medical supply and packaging necessary for administration and the waste treatment. Abbreviations: API: Active Pharmaceutical Ingredient.

| **Impact category** | **Unit** | **Total** | **API** | **Galenic** | **Medical supply** | **Waste treatment** |
| --- | --- | --- | --- | --- | --- | --- |
| Global warming | kg CO2 eq | 5,29E-01 | 4,37E-03 | 8,05E-04 | 3,02E-01 | 2,22E-01 |
| Stratospheric ozone depletion | kg CFC11 eq | 3,37E-07 | 1,63E-09 | 5,91E-10 | 1,95E-07 | 1,40E-07 |
| Ionizing radiation | kBq Co-60 eq | 1,41E-01 | 1,75E-04 | 7,13E-05 | 1,40E-01 | 2,73E-04 |
| Ozone formation, Human health | kg NOx eq | 7,80E-04 | 7,85E-06 | 6,05E-06 | 6,19E-04 | 1,47E-04 |
| Fine particulate matter formation | kg PM2.5 eq | 4,21E-04 | 5,25E-06 | 2,99E-06 | 3,83E-04 | 3,01E-05 |
| Ozone formation, Terrestrial ecosystems | kg NOx eq | 8,20E-04 | 8,22E-06 | 6,11E-06 | 6,56E-04 | 1,50E-04 |
| Terrestrial acidification | kg SO2 eq | 9,36E-04 | 1,16E-05 | 7,95E-06 | 8,40E-04 | 7,59E-05 |
| Freshwater eutrophication | kg P eq | 1,98E-04 | 5,75E-06 | 4,04E-07 | 1,26E-04 | 6,61E-05 |
| Marine eutrophication | kg N eq | 2,36E-05 | 3,72E-06 | 2,72E-08 | 1,68E-05 | 3,01E-06 |
| Terrestrial ecotoxicity | kg 1,4-DCB | 2,07E+00 | 1,21E-02 | 7,57E-03 | 1,96E+00 | 9,20E-02 |
| Freshwater ecotoxicity | kg 1,4-DCB | 7,77E-02 | 1,44E-04 | 7,99E-05 | 1,61E-02 | 6,14E-02 |
| Marine ecotoxicity | kg 1,4-DCB | 1,03E-01 | 1,90E-04 | 1,05E-04 | 2,26E-02 | 8,04E-02 |
| Human carcinogenic toxicity | kg 1,4-DCB | 2,51E-02 | 3,09E-04 | 9,30E-05 | 1,67E-02 | 8,00E-03 |
| Human non-carcinogenic toxicity | kg 1,4-DCB | 1,66E+00 | 3,14E-03 | 1,46E-03 | 5,28E-01 | 1,13E+00 |
| Land use | m2a crop eq | 4,13E-02 | 6,96E-05 | 2,96E-05 | 4,10E-02 | 2,81E-04 |
| Mineral resource scarcity | kg Cu eq | 1,57E-03 | 9,22E-06 | 6,79E-06 | 1,49E-03 | 6,86E-05 |
| Fossil resource scarcity | kg oil eq | 1,21E-01 | 1,45E-03 | 2,17E-04 | 1,15E-01 | 4,22E-03 |
| Water consumption | m3 | 9,34E-03 | 6,92E-05 | 1,49E-04 | 8,80E-03 | 3,21E-04 |

**Supplementary Table 8. Detailed environmental impacts across 18 different categories for one dose of ciprofloxacin administered IV (400 mg) - alternative scenario: all materials are recycled after use.** Impacts are subcategorized into their four components: related to the API, the galenic formulation, the medical supply and packaging necessary for administration and the waste treatment. Abbreviations: API: Active Pharmaceutical Ingredient.

| **Impact category** | **Unit** | **Total** | **API** | **Galenic** | **Medical supply** | **Waste treatment** |
| --- | --- | --- | --- | --- | --- | --- |
| Global warming | kg CO2 eq | 7,34E-01 | 4,37E-03 | 8,05E-04 | 4,71E-01 | 2,58E-01 |
| Stratospheric ozone depletion | kg CFC11 eq | 9,38E-07 | 1,63E-09 | 5,91E-10 | 7,83E-07 | 1,53E-07 |
| Ionizing radiation | kBq Co-60 eq | 8,68E-02 | 1,75E-04 | 7,13E-05 | 1,95E-01 | -1,09E-01 |
| Ozone formation, Human health | kg NOx eq | 1,00E-03 | 7,85E-06 | 6,05E-06 | 9,90E-04 | -4,18E-06 |
| Fine particulate matter formation | kg PM2.5 eq | 5,43E-04 | 5,25E-06 | 2,99E-06 | 5,94E-04 | -5,95E-05 |
| Ozone formation, Terrestrial ecosystems | kg NOx eq | 1,05E-03 | 8,22E-06 | 6,11E-06 | 1,05E-03 | -1,38E-05 |
| Terrestrial acidification | kg SO2 eq | 1,23E-03 | 1,16E-05 | 7,95E-06 | 1,39E-03 | -1,74E-04 |
| Freshwater eutrophication | kg P eq | 3,02E-04 | 5,75E-06 | 4,04E-07 | 1,93E-04 | 1,02E-04 |
| Marine eutrophication | kg N eq | 1,07E-04 | 3,72E-06 | 2,72E-08 | 9,56E-05 | 7,18E-06 |
| Terrestrial ecotoxicity | kg 1,4-DCB | 2,05E+00 | 1,21E-02 | 7,57E-03 | 2,47E+00 | -4,39E-01 |
| Freshwater ecotoxicity | kg 1,4-DCB | 1,49E-01 | 1,44E-04 | 7,99E-05 | 2,35E-02 | 1,25E-01 |
| Marine ecotoxicity | kg 1,4-DCB | 1,96E-01 | 1,90E-04 | 1,05E-04 | 3,22E-02 | 1,63E-01 |
| Human carcinogenic toxicity | kg 1,4-DCB | 3,30E-02 | 3,09E-04 | 9,30E-05 | 2,42E-02 | 8,42E-03 |
| Human non-carcinogenic toxicity | kg 1,4-DCB | 2,88E+00 | 3,14E-03 | 1,46E-03 | 7,48E-01 | 2,13E+00 |
| Land use | m2a crop eq | 2,96E-02 | 6,96E-05 | 2,96E-05 | 6,32E-02 | -3,38E-02 |
| Mineral resource scarcity | kg Cu eq | 1,19E-03 | 9,22E-06 | 6,79E-06 | 2,10E-03 | -9,30E-04 |
| Fossil resource scarcity | kg oil eq | 1,29E-01 | 1,45E-03 | 2,17E-04 | 1,76E-01 | -4,79E-02 |
| Water consumption | m3 | 1,44E-02 | 6,92E-05 | 1,49E-04 | 1,99E-02 | -5,76E-03 |

**Supplementary Table 9. Detailed environmental impacts across 18 different categories for one dose of ciprofloxacin administered orally (500 mg) - main scenario.** Impacts are subcategorized into their four components: related to the API, the galenic formulation, the medical supply and packaging necessary for administration and the waste treatment. Abbreviations: API: Active Pharmaceutical Ingredient.

| **Impact category** | **Unit** | **Total** | **API** | **Galenic** | **Medical supply** | **Waste treatment** |
| --- | --- | --- | --- | --- | --- | --- |
| Global warming | kg CO2 eq | 1,26E-02 | 5,46E-03 | 1,03E-03 | 4,59E-03 | 1,52E-03 |
| Stratospheric ozone depletion | kg CFC11 eq | 7,20E-09 | 2,04E-09 | 1,97E-09 | 2,45E-09 | 7,33E-10 |
| Ionizing radiation | kBq Co-60 eq | 3,64E-03 | 2,18E-04 | 6,79E-04 | 2,73E-03 | 1,27E-05 |
| Ozone formation, Human health | kg NOx eq | 2,72E-05 | 9,81E-06 | 5,69E-06 | 1,09E-05 | 8,46E-07 |
| Fine particulate matter formation | kg PM2.5 eq | 1,64E-05 | 6,56E-06 | 2,87E-06 | 6,65E-06 | 3,36E-07 |
| Ozone formation, Terrestrial ecosystems | kg NOx eq | 2,84E-05 | 1,03E-05 | 5,76E-06 | 1,15E-05 | 8,54E-07 |
| Terrestrial acidification | kg SO2 eq | 3,93E-05 | 1,45E-05 | 9,50E-06 | 1,46E-05 | 6,93E-07 |
| Freshwater eutrophication | kg P eq | 1,10E-05 | 7,18E-06 | 7,22E-07 | 2,61E-06 | 4,65E-07 |
| Marine eutrophication | kg N eq | 5,47E-06 | 4,65E-06 | 4,55E-07 | 3,47E-07 | 2,00E-08 |
| Terrestrial ecotoxicity | kg 1,4-DCB | 6,00E-02 | 1,51E-02 | 5,57E-03 | 3,88E-02 | 5,99E-04 |
| Freshwater ecotoxicity | kg 1,4-DCB | 6,24E-04 | 1,81E-04 | 8,27E-05 | 3,07E-04 | 5,38E-05 |
| Marine ecotoxicity | kg 1,4-DCB | 8,53E-04 | 2,37E-04 | 1,11E-04 | 4,31E-04 | 7,30E-05 |
| Human carcinogenic toxicity | kg 1,4-DCB | 8,28E-04 | 3,87E-04 | 9,13E-05 | 3,12E-04 | 3,74E-05 |
| Human non-carcinogenic toxicity | kg 1,4-DCB | 1,72E-02 | 3,93E-03 | 2,32E-03 | 1,01E-02 | 8,15E-04 |
| Land use | m2a crop eq | 1,41E-03 | 8,70E-05 | 3,29E-04 | 9,91E-04 | 3,73E-06 |
| Mineral resource scarcity | kg Cu eq | 5,32E-05 | 1,15E-05 | 1,19E-05 | 2,91E-05 | 6,66E-07 |
| Fossil resource scarcity | kg oil eq | 3,97E-03 | 1,81E-03 | 2,82E-04 | 1,84E-03 | 3,55E-05 |
| Water consumption | m3 | 3,11E-04 | 8,65E-05 | 4,72E-05 | 1,73E-04 | 4,04E-06 |

**Supplementary Table 10. Detailed environmental impacts across 18 different categories for one dose of ciprofloxacin administered orally (500 mg) - alternative scenario: all recyclable materials are recycled after use.** Impacts are subcategorized into their four components: related to the API, the galenic formulation, the medical supply and packaging necessary for administration and the waste treatment. Abbreviations: API: Active Pharmaceutical Ingredient.

| **Impact category** | **Unit** | **Total** | **API** | **Galenic** | **Medical supply** | **Waste treatment** |
| --- | --- | --- | --- | --- | --- | --- |
| Global warming | kg CO2 eq | 1,08E-02 | 5,46E-03 | 1,03E-03 | 4,59E-03 | -2,54E-04 |
| Stratospheric ozone depletion | kg CFC11 eq | 6,08E-09 | 2,04E-09 | 1,97E-09 | 2,45E-09 | -3,84E-10 |
| Ionizing radiation | kBq Co-60 eq | 2,13E-03 | 2,18E-04 | 6,79E-04 | 2,73E-03 | -1,50E-03 |
| Ozone formation, Human health | kg NOx eq | 2,35E-05 | 9,81E-06 | 5,69E-06 | 1,09E-05 | -2,84E-06 |
| Fine particulate matter formation | kg PM2.5 eq | 1,47E-05 | 6,56E-06 | 2,87E-06 | 6,65E-06 | -1,39E-06 |
| Ozone formation, Terrestrial ecosystems | kg NOx eq | 2,45E-05 | 1,03E-05 | 5,76E-06 | 1,15E-05 | -2,98E-06 |
| Terrestrial acidification | kg SO2 eq | 3,50E-05 | 1,45E-05 | 9,50E-06 | 1,46E-05 | -3,61E-06 |
| Freshwater eutrophication | kg P eq | 1,02E-05 | 7,18E-06 | 7,22E-07 | 2,61E-06 | -2,93E-07 |
| Marine eutrophication | kg N eq | 5,36E-06 | 4,65E-06 | 4,55E-07 | 3,47E-07 | -9,64E-08 |
| Terrestrial ecotoxicity | kg 1,4-DCB | 5,11E-02 | 1,51E-02 | 5,57E-03 | 3,88E-02 | -8,26E-03 |
| Freshwater ecotoxicity | kg 1,4-DCB | 4,59E-04 | 1,81E-04 | 8,27E-05 | 3,07E-04 | -1,12E-04 |
| Marine ecotoxicity | kg 1,4-DCB | 6,26E-04 | 2,37E-04 | 1,11E-04 | 4,31E-04 | -1,54E-04 |
| Human carcinogenic toxicity | kg 1,4-DCB | 6,91E-04 | 3,87E-04 | 9,13E-05 | 3,12E-04 | -9,97E-05 |
| Human non-carcinogenic toxicity | kg 1,4-DCB | 1,20E-02 | 3,93E-03 | 2,32E-03 | 1,01E-02 | -4,34E-03 |
| Land use | m2a crop eq | 5,52E-04 | 8,70E-05 | 3,29E-04 | 9,91E-04 | -8,55E-04 |
| Mineral resource scarcity | kg Cu eq | 3,80E-05 | 1,15E-05 | 1,19E-05 | 2,91E-05 | -1,45E-05 |
| Fossil resource scarcity | kg oil eq | 3,42E-03 | 1,81E-03 | 2,82E-04 | 1,84E-03 | -5,14E-04 |
| Water consumption | m3 | 2,19E-04 | 8,65E-05 | 4,72E-05 | 1,73E-04 | -8,78E-05 |

**Supplementary Figure 3. Comparative environmental impacts across 18 different categories for one dose of ciprofloxacin administered orally (500 mg) or IV (400 mg) (main scenario).** Impacts are subcategorized into their four components: related to the API, the galenic formulation, the medical supply and packaging necessary for administration and the waste treatment. Abbreviations: API: Active Pharmaceutical Ingredient; PO: per os administration; IV intravenous administration.


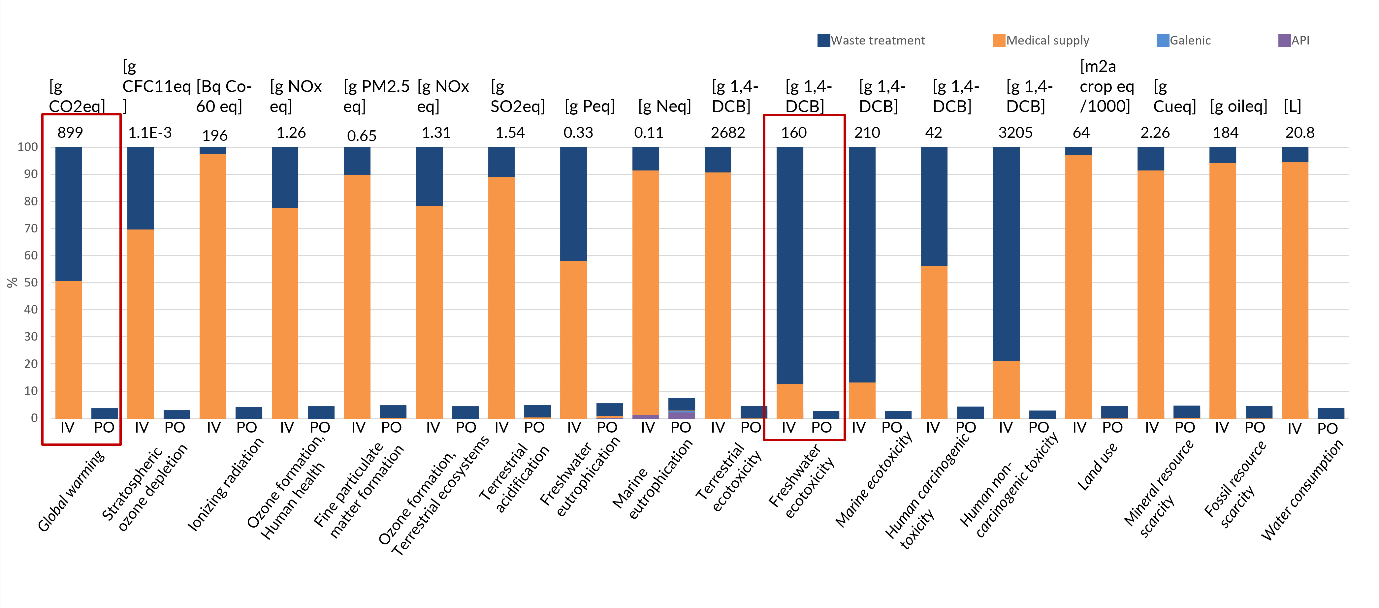


### References

1. Yang K, Lv B, Shen H et al. Life cycle assessment of pharmaceuticals: the ciprofloxacin hydrochloride case. *The International Journal of Life Cycle Assessment* 2021; **26**: 64-75.

2. Sharma RK, Sarkar P, Singh H. Assessing the sustainability of a manufacturing process using life cycle assessment technique—a case of an Indian pharmaceutical company. *Clean Technologies and Environmental Policy* 2020; **22**: 1269-84.

3. Sharma RK, Raju G, Sarkar P et al. Comparing the environmental impacts of paracetamol dosage forms using life cycle assessment. *Environment, Development and Sustainability* 2022; **24**: 12446-66.

4. USEtox. <https://usetox.org/> (28/08/2024.

5. Ortiz de García S, García-Encina PA, Irusta-Mata R. The potential ecotoxicological impact of pharmaceutical and personal care products on humans and freshwater, based on USEtox™ characterization factors. A Spanish case study of toxicity impact scores. *Science of The Total Environment* 2017; **609**: 429-45.

6. Davies JF, McAlister S, Eckelman MJ et al. Environmental and financial impacts of perioperative paracetamol use: a multicentre international life-cycle assessment. *Br J Anaesth* 2024; **133**: 1439-48.
